# The novel β2-selective proteasome inhibitor LU-102 decreases phosphorylation of I kappa B and induces highly synergistic cytotoxicity in combination with ibrutinib in multiple myeloma cells

**DOI:** 10.1007/s00280-015-2801-0

**Published:** 2015-06-23

**Authors:** Johannes Kraus, Marianne Kraus, Nora Liu, Lenka Besse, Jürgen Bader, Paul P. Geurink, Gerjan de Bruin, Alexei F. Kisselev, Herman Overkleeft, Christoph Driessen

**Affiliations:** Experimental Oncology and Hematology, Department of Oncology and Hematology, Kantonsspital St. Gallen, 9007 St. Gallen, Switzerland; Gorlaeus Laboratories, Leiden Institute of Chemistry and Netherlands Proteomics Centre, Einsteinweg 55, 2333 CC Leiden, The Netherlands; Department of Pharmacology and Toxicology, Norris Cotton Cancer Center, Geisel School of Medicine at Dartmouth, 1 Medical Center Drive HB7936, Lebanon, NH 03756 USA

**Keywords:** Protease inhibitors, Myeloma therapy, Signal transduction, Drug resistance

## Abstract

**Purpose:**

Proteasome-inhibiting drugs (PI) are gaining importance in hematologic oncology. The proteasome carries three proteolytically active subunits (β1, β2, β5). All established PI (bortezomib and carfilzomib), as well as experimental drugs in the field (dalanzomib, oprozomib, and ixazomib), by design target the rate-limiting β5 subunit. It is unknown whether β2-selective proteasome inhibition can also be exploited toward anticancer treatment. Combining PI with the pan B-cell-directed Bruton tyrosine kinase inhibitor ibrutinib appears a natural option for future improved treatment of multiple myeloma (MM) and B-cell lymphomas. However, bortezomib induces phosphorylation of IκB and activation of NF-κB in MM cells, while ibrutinib inhibits the IκB/NF-κB axis, suggesting antagonistic signaling. A β2-selective proteasome inhibitor may lack such antagonistic signaling effects.

**Methods:**

We recently introduced LU-102, the first β2-selective PI available for preclinical testing. We here compare bortezomib with carfilzomib and LU-102 in MM and MCL in vitro with regard to their effects on pIκB/NF-κB signaling and their cytotoxic activity in combination with ibrutinib.

**Results:**

LU-102 reduced phosphorylation of IκB, in contrast to bortezomib and carfilzomib, and was a superior inhibitor of NF-κB activation in MM cells. This translated into highly synergistic cytotoxicity between LU-102 and ibrutinib, which was able to overcome BTZ resistance and CFZ resistance. By contrast, BTZ lacked consistent synergistic cytotoxicity with ibrutinib.

**Conclusion:**

Ibrutinib is highly synergistic with β2-selective proteasome inhibition against MM and MCL in vitro. Novel β2-selective proteasome inhibitors may be exploited to overcome bortezomib/carfilzomib resistance and boost the activity of BTK inhibitors against B-cell-derived malignancies.

## Introduction

Treatment with proteasome inhibitors (PI) has become a backbone of therapy for multiple myeloma (MM) [1] and mantle cell lymphoma (MCL). This has established the proteasome as a molecular target and the manipulation of protein homeostasis as a therapeutic principle in cancer. However, bortezomib (BTZ), the first in class approved PI, is lacking significant single-agent activity in other B-cell-derived malignancies, so that several combination treatments are under clinical investigation [2]. In addition, resistance to BTZ almost inevitably occurs in MM and MCL patients during the course of the disease [1], underscoring the need to improve the activity of PI-based treatments.

The proteasome is a multicatalytic multiprotein protease complex responsible for the majority of protein destruction in eukaryotic cells [3]. Its proteolytic activity is mediated by three distinct active sites in the β1, β2, and β5 subunits that hydrolyze substrates with distinct substrate specificities (caspase-like, trypsin-like, and chymotrypsin-like). BTZ is a peptide-based, reversible inhibitor of the β5-type, and to a lesser extent the β1-type proteasome subunits, that was developed based on the finding that the β5 active site mediates the rate-limiting activity for proteasomal proteolysis [1]. Several second-generation, peptide-based, irreversible PI are in clinical development, including the FDA-approved carfilzomib (CFZ), as well as ixazomib [4] and oprozomib [5], which all by design target the β5 proteasome activity. The clinical activity of CFZ in BTZ-refractory MM is low and in the 20 % range [6].

Continuing progress in resolving the three-dimensional structure of the proteasome in complex with PI has facilitated the development of PI that selectively target the non-β5 proteasome subunits, i.e., β2, β1 and the immuno-proteasome. The β2 proteasome activity has been identified as critical factor that modulates the cytotoxicity of β5-targeted proteasome inhibitors [7–9], corresponding with the finding that β2 proteasome activity is upregulated in BTZ-refractory cells [10]. We have recently developed LU-102, the first irreversible, cell-permeable, β2-selective PI as a chemical lead for preclinical development [8], which is currently being tested in combination with the approved PI to overcome PI resistance in preclinical models. The potential of LU-102 for therapeutic use in combination with non-PI anticancer agents remains to be defined.

B-cell-derived malignancies represent a natural target population for the development of PI-based combination therapies, such as CLL and mantle cell lymphoma [11, 12]. In addition, PI-refractory myeloma in vivo accumulates features of immature B cells [13], also implementing the use of pan B-cell-directed targeted agents against advanced MM. Hence, combining proteasome inhibitor therapy with a broadly active B-cell-targeting drug is a promising concept to further exploit the cytotoxic anticancer activity of PI, in particular in MCL, CLL, and BTZ-refractory MM.

The Bruton tyrosin kinase (BTK) is a non-receptor tyrosine kinase expressed throughout the entire B-cell differentiation [14], which plays a key role in B-cell development and function [15]. BTK signals through phosphorylation of PLC-γ, leading to phosphorylation of IκB and activation of the NF-κB signaling pathway, and also induces MAPK and AKT signaling. MAPK, STAT3, and in particular NF-κB, are critical signaling pathways for MM cell survival [16]. Ibrutinib is approved as first in class BTK inhibitor in MCL, chronic lymphatic leukemia, and Waldenström’s disease, and has shown clinical activity in MM and other B-cell-derived neoplasms. Combining ibrutinib with PI is currently explored using BTZ or CFZ, respectively, in MM and MCL.

The major signaling pathway of BTK as well as strong downstream signaling effects of BTZ converge in the canonical NF-κB pathway [17]: IκB is phosphorylated downstream of active BTK, generating p-IκB which is a target for ubiquitination and proteasomal disposal, liberating the transactivating activity of NF-κB. Hence, the cytotoxic activity of ibrutinib is mediated via a decrease in p-IκB, leading to decreased NF-κB activity. By contrast, BTZ has been shown to increase p-IκB and induce NF-κB activity in MM cells [18]. This implies that combination therapies between BTZ and BTK inhibitors may have opposing effects on NF-κB signaling, which would be expected to limit their activity. However, also negative regulation of the NF-kB pathways by BTZ and induction of IkBα degradation have been shown [19–21]. The mechanism of the stabilization of p-IκB and NF-κB activity by BTZ in MM is poorly understood, but may involve off-target effects of BTZ, which is known to inhibit also lysosomal serine proteases such as cathepsin G and potentially other proteases [22]. Novel irreversible PI such as CFZ and LU-102 are more selective for the proteasome and lack such off-target activity, so that they may be more suitable combination partners for ibrutinib to treat MM. The aim of the current study was to compare ibrutinib in combination with BTZ to combinations of ibrutinib with either CFZ or LU-102 with respect to the resulting effects on BTK signaling and cytotoxicity in MM cells, including BTZ-resistant myeloma. This aims at providing a preclinical rationale to select either BTZ or CFZ as combination partner for ibrutinib in MM clinical trials, and in addition shall allow to further explore a potential use of β2-selective proteasome inhibitors as combination partners in targeted therapies, in particular in the MM field.

## Methods and materials

### Cells and inhibitors

Human myeloma cell lines RPMI 8226, LP-1, AMO-1, U-266, MM.1S, MM.1R, human mantle cell lymphoma cell lines Granta-519 and Jeko-1, and the acute myeloid leukemia cell line THP-1 were obtained from ATCC, human myeloma cell lines INA-6, JK-6, L363 from M. Gramatzki, Kiel, and were maintained in FCS-supplemented RPMI 1640 medium with gentamycin. INA-6 is IL-6 dependent which was used at 500 U/ml. AMO-BTZ/CFZ cells were adapted to proteasome inhibitor-containing culture conditions from the AMO-1 parental line as described [10]. The proteasome inhibitors bortezomib, carfilzomib, and LU-102 [8] were synthesized at the Leiden Institute of Chemistry, and ibrutinib was obtained from Pharmacyclics. The activity-based ibrutinib probe was synthesized and used as described recently [27].

### MTT assay, Western blot, Western blot quantification, antibodies

CellTiter 96^®^ AQueous One Solution cell proliferation assay (Promega) was used to determine cell viability in MTT assays. Mean values from quadruplicate samples of one representative experiment representing at least three independent experiments are presented. SDS-PAGE and Western blot was performed as described [10]. Western blots were quantified using Bio 1D software (Vilber Lourmat); Fluorescence signals of DMSO-treated cells were considered baseline levels. Anti-BTK (Tyr223), anti-p-BTK (pTyr223), anti-p-65, and anti-cleaved caspase 3, 7, 9, anti-STAT3, and anti-p-STAT3 antibodies were purchased from Cell Signalling Technology (Boston, USA), anti-IκBα and anti-p-IκBα from Becton–Dickinson (Heidelberg, Germany), anti-cleaved PARP from Promega (Madison, USA), anti-GAPDH and anti-β-actin from Sigma (St. Louis, USA), and anti-polyubiquitinated proteins from Viva Bioscience (Exeter, UK).

### Determination of proteasome activity by active site labeling

The covalent, proteasome-specific affinity probe Bodipy TMR-Ahx3L3VS (MV-151) was synthesized and used as described [23]. Both the constitutive and the immune-proteasome subunits were irreversibly labeled by MV151 in intact cells and resolved after cell lysis by SDS-PAGE. Proteasome subunit-specific fluorescence signals were in-gel measured with the Fusion FX7 (Vilber Lourmat) and quantified using Bio 1D software.

### RNA extraction and real-time PCR

RNA was extracted from 5 × 10^6^ cells using phenol–chloroform method. Reverse transcription was performed using the High-Capacity cDNA Reverse Transcription Kit with RNAse Inhibitor (Applied Biosystems). Real-time PCR primers for GAPDH and BTK were purchased from Qiagen. Relative quantitative real-time PCR used SYBR green technology (Roche) on cDNA generated from the reverse transcription of purified RNA. After preamplification (95 °C for 10 min), the PCRs were amplified for 45 cycles (95 °C for 10 s, 55 °C for 20 s and 72 °C for 20 s) on a LightCycler^®^ 480 (Roche). Each mRNA expression was normalized against GAPDH mRNA expression using the standard curve method.

### Patient cells

The primary myeloma cell samples were obtained after written informed consent and approval by the independent ethics review board, in accordance with ICH-GCP and local regulations. Malignant plasma cells were retrieved by bone marrow aspiration from patients with multiple myeloma progressing under bortezomib-containing therapy (BTZ-resistant) or responding to bortezomib (BTZ-sensitive), based on IMWG criteria. The purity of the cell sample was >80 % myeloma cells after Ficoll separation, as assessed by morphology. Cells were cultured in FCS-supplemented RPMI 1640 medium with gentamycin.

### Statistical analysis

Unless stated otherwise, one representative experiment out of at least three independent experiments is shown; for MTT assays, mean values from quadruplicate samples are represented. Error bars indicate the standard deviation between the individual experiments or samples. Synergism between ibrutinib and the different proteasome inhibitors was calculated using MTT assays in conjunction with the combination index described by Chou et al. [24]. A combination Index <0.8 indicates synergism, >1 indicates antagonism. The statistical significance was calculated using student’s *t* test.

## Results

### BTK expression and ibrutinib-mediated cytotoxicity in MM cell lines

**Fig. 1:**
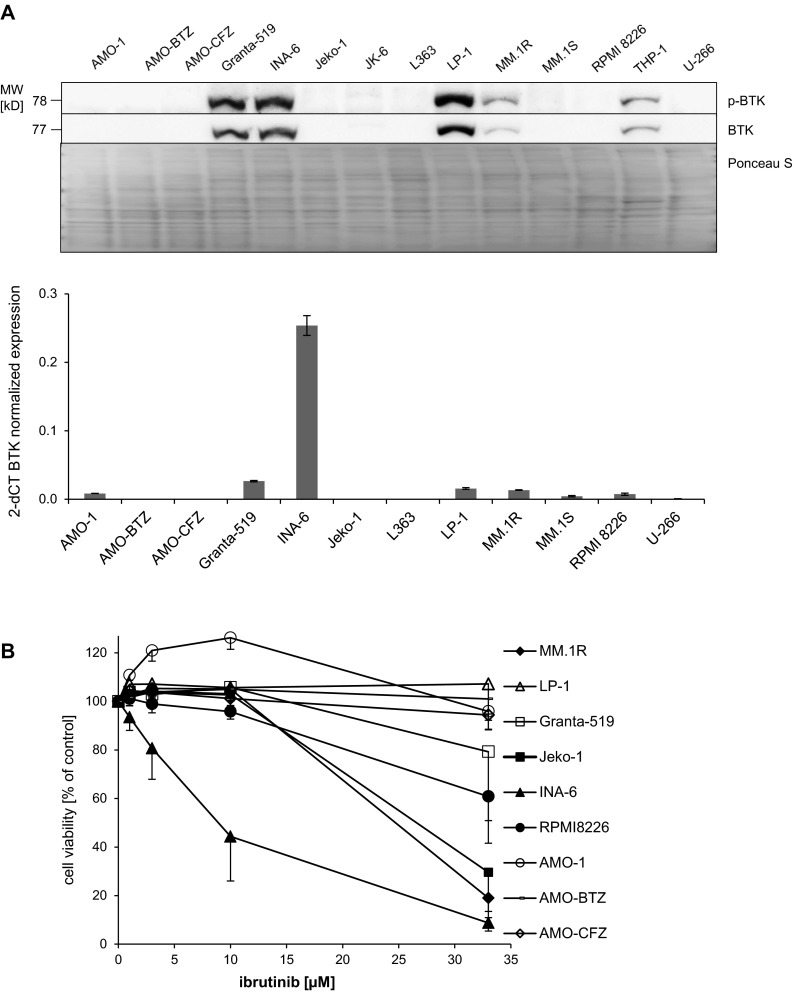
BTK expression and ibrutinib-mediated cytotoxicity in MM cell lines. **a** *Upper panel* MM cell lines (AMO-1, AMO-BTZ, AMO-CFZ, INA-6, JK-6, L363, LP-1 MM.1R, MM.1S, RPMI 8226 and U-266), MCL cell lines (Granta-519 and Jeko-1), and AML cell line (THP-1) were analyzed with respect to protein expression of BTK. After cell lysis, equal amounts of protein were resolved by SDS-PAGE, and Western blots against BTK and activated BTK (p-BTK) were performed. Ponceau S staining of the same PVDF membrane that was used for the blots confirms equal protein contents between lanes. *Lower panel* The same cell lines were analyzed for BTK mRNA expression by real-time PCR. Results are expressed in relation to mRNA for GAPDH. **b** MM cell lines (MM.1R, LP-1, INA-6, RPMI 8226, AMO-1, AMO-BTZ and AMO-CFZ) and MCL cell lines (Granta-519 and Jeko-1) were incubated with ibrutinib at indicated concentrations for 48 h and cell viability was assessed by MTT proliferation assay

We analyzed a panel of MM and mantle cell lymphoma (MCL) cell lines with respect to protein and mRNA expression of BTK and p-BTK, respectively, and correlated the results with the cytotoxic effect of ibrutinib in vitro. Consistent with published data [25, 26], we found sizable BTK protein expression in the MM cell lines INA-6, LP-1, and to a lesser extent in MM.1R cells, in contrast to the remaining MM cell lines (AMO-1, AMO-BTZ, AMO-CFZ, JK-6, L363, MM.1S, RPMI 8226 and U-266; Fig. 1a). The mRNA transcription levels for BTK only poorly correlated with the respective protein expression, also in agreement with earlier studies [25]. Interestingly, the sensitivity of MM and MCL cell lines for ibrutinib-induced cytotoxicity also only poorly reflected the protein expression levels of p-BTK in the individual cell lines (Fig. 1b). Because the majority of primary human MM cell samples express p-BTK protein and are sensitive to cytotoxic treatment with ibrutinib 10 μM in vitro [26], we selected INA-6 MM cells as a suitable model system to study the effects of ibrutinib in combination with proteasome inhibitors on MM cell lines in vitro.

### Ibrutinib reduces p-IκB levels and lacks a direct effect on proteasome activity in MM cell lines

**Fig. 2:**
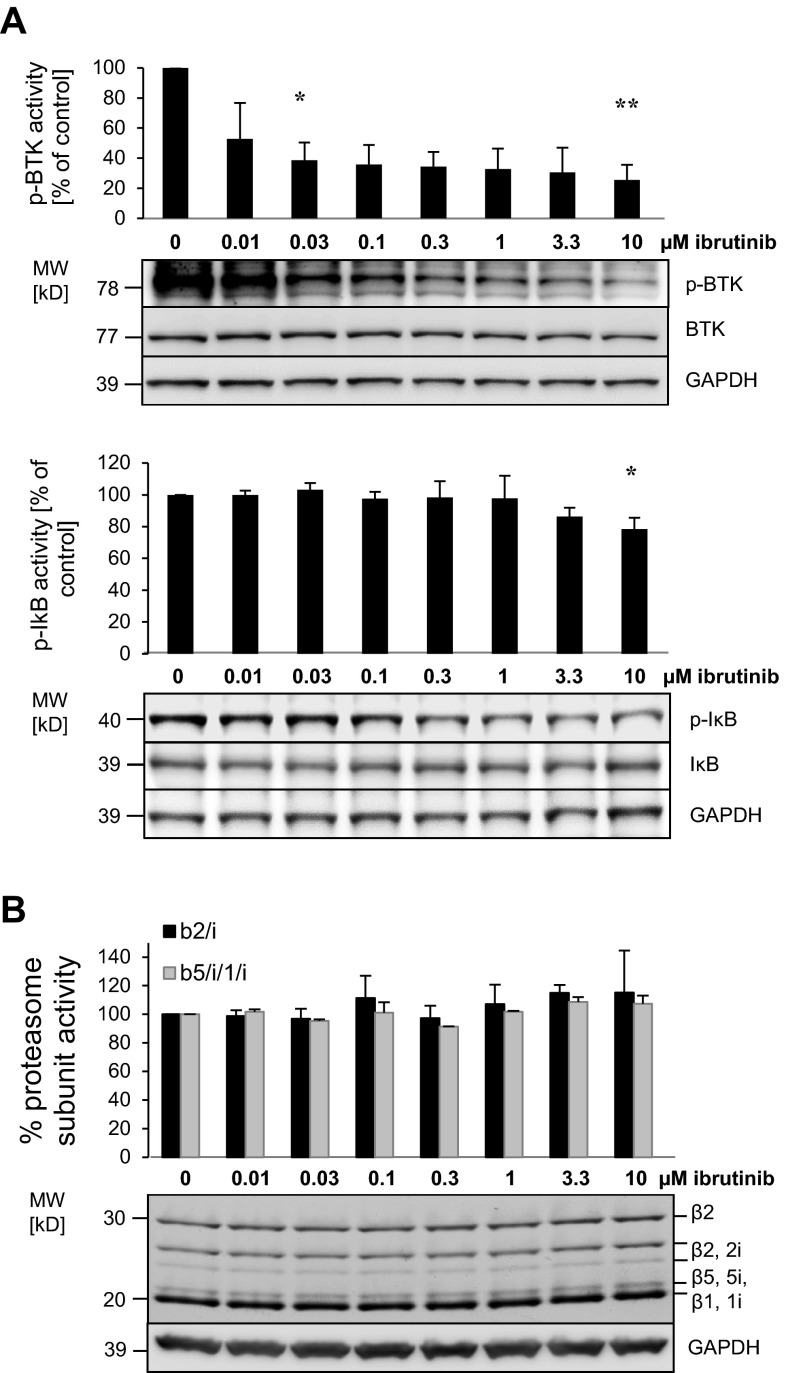
Molecular effects of ibrutinib on the target proteins p-BTK/BTK, the downstream p-IκB/IκB activation and proteasome subunit activity. **a** *Upper panel* INA-6 cells were incubated with increasing ibrutinib concentrations (0–10 µM) for 4 h, and p-BTK and BTK proteins were assessed by Western blot. The bar graph illustrates the quantitative comparison of the fluorescence signals retrieved for p-BTK protein at the respective ibrutinib concentrations, relative to baseline (DMSO-treated). *Lower panel* INA-6 cells were incubated with increasing ibrutinib concentrations (0–10 µM) for 8 h, before IκB and activated IκB (p-IκB) proteins were determined by Western blot and quantified as described above. For a statistically significant quantitative difference from baseline, *p < 0.05; **p < 0.01. **b** After incubation with increasing ibrutinib concentrations (0–10 µM), active proteasome subunits in INA-6 cells were affinity-labeled using the fluorescent, pan-proteasome reactive, cell-permeable probe MV-151 for 1 h. After resolution by SDS-PAGE, the fluorescence signals representing active proteasome β1, β2, β5 polypeptides and the respective immuno-proteasome species β1i, β2i, β5i were visualized using a fluorescent reader and quantitated. Conventional Western blotting against GAPDH demonstrates equal protein load of the samples. The bar graph above illustrates the quantitative comparison of the specific fluorescence signals detected for the proteasomal β1(i)/β5(i) and β2(i) activities, relative to DMSO-treated baseline

We next assessed the molecular effects of ibrutinib on the p-BTK/p-IκB signaling cascade as well as on the proteasome activity in INA-6 cells. As expected, ibrutinib inhibited the p-BTK expression in a dose-dependent manner already at nanomolar concentrations (Fig. 2a). Likewise, a dose-dependent reduction in p-IκB expression consistent with the known effect of ibrutinib on BTK signaling was observed, starting at high nanomolar drug levels. As expected, ibrutinib had no direct effect on the activity of the proteasomal β1, β2, or β5 subunits, as visualized by the cell-permeable, pan-proteasome-selective, activity-based probe MV151 that irreversibly targets the active constitutive and immuno-proteasome subunits in situ and allows their direct quantification by fluorescence detection (Fig. 2b).

### The β2-selective proteasome inhibitor LU-102 decreases p-IκB expression, in contrast to bortezomib or carfilzomib

**Fig. 3:**
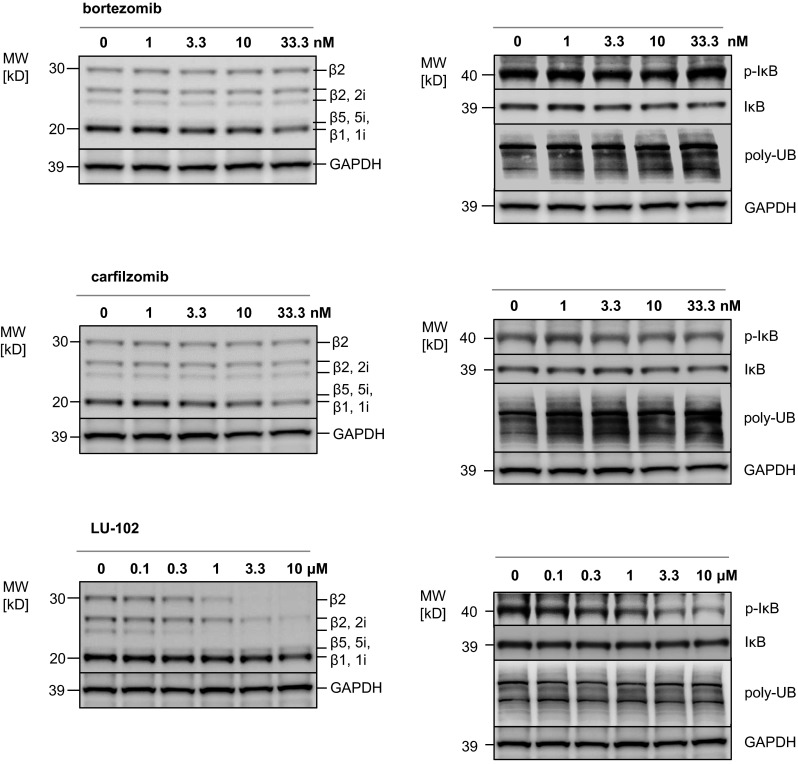
Molecular effects of bortezomib, carfilzomib, and the β2-specific proteasome inhibitor LU-102 on proteasome subunit activities, the p-IκB/IκB ratio and polyubiquitinated protein. *Left panels* After incubation with increasing proteasome inhibitor concentrations (bortezomib, carfilzomib: 0–33.3 nM; LU-102: 0–10 µM), active proteasome subunits in INA-6 cells were affinity-labeled using the cell-permeable probe MV-151, and visualized as before. *Right panels* INA-6 cells were incubated with the respective proteasome inhibitors, as before, followed by assessment of p-IκB, IκB, and polyubiquitinated proteins (poly-UB) by Western blots. One representative of two experiments performed

The cytotoxic activity of ibrutinib is transmitted via reduction in p-IκB levels, while in contrast BTZ has been shown to moderately increase p-IκB in MM cells [18], which would predict to limit the synergistic activity of both drugs. We therefore compared the effect of BTZ on p-IκB with that of CFZ, as well as the β2-selective peptide vinylsulfone-type proteasome inhibitor LU-102 [8]. INA-6 MM cells were incubated with the respective proteasome inhibitors in a concentration range that included the IC50 for each inhibitor, and their differential effects on proteasome activity, the accumulation of polyubiquitinated cellular protein, and p-IκB/IκB protein expression were visualized (Fig. 3). In BTZ-treated cells, we observed a concentration-dependent, β1-/β5-selective reduction in proteasome activity that was accompanied by a respective increase in polyubiquitinated protein. Also in agreement with published data, BTZ treatment led to a slight decrease in IκB, while p-IκB expression increased (Fig. 3, upper panel), resulting in an increased p-IκB/IκB ratio. CFZ treatment did likewise reduce the β1/β5 proteasome activity signal with a concomitant increase in polyubiquitinated protein, comparable to BTZ. CFZ treatment at higher concentrations (10 nM and higher) in addition resulted in a small, but reproducible reduction in β2 activity, unlike BTZ. The ratio of p-IκB/IκB expression likewise increased under CFZ treatment (Fig. 3, middle panel). In contrast to both approved proteasome inhibitors, LU-102 specifically targeted the β2-/2i-type proteasome activity, without affecting the activity signals for β1/β5 subunits (Fig. 3, lower panel). An increase in polyubiquitinated protein was not observed with LU-102 treatment, suggesting less effective quantitative reduction in protein degradation during β2-selective proteasome inhibition. Surprisingly, and in contrast to BTZ and CFZ, LU-102 treatment resulted in a concentration-dependent decrease in p-IκB levels, so that a significant decrease in the ratio of p-IκB/IκB expression was observed in INA-6 cells after LU-102 treatment. Thus, the β2-selective proteasome inhibitor LU-102 decreases cellular p-IκB levels and the p-IκB/IκB ratio and therefore may support the signaling cascade triggered by ibrutinib, in contrast to the β1/β5-selective proteasome inhibitors CFZ and BTZ that stabilize p-IκB levels and increase the p-IκB/IκB ratio. We speculated that LU-102 may result in superior synergistic cytotoxic activity in combination with ibrutinib, compared to BTZ or CFZ.

### Ibrutinib in combination with LU-102 shows superior synergistic cytotoxicity against MM cells

**Fig. 4:**
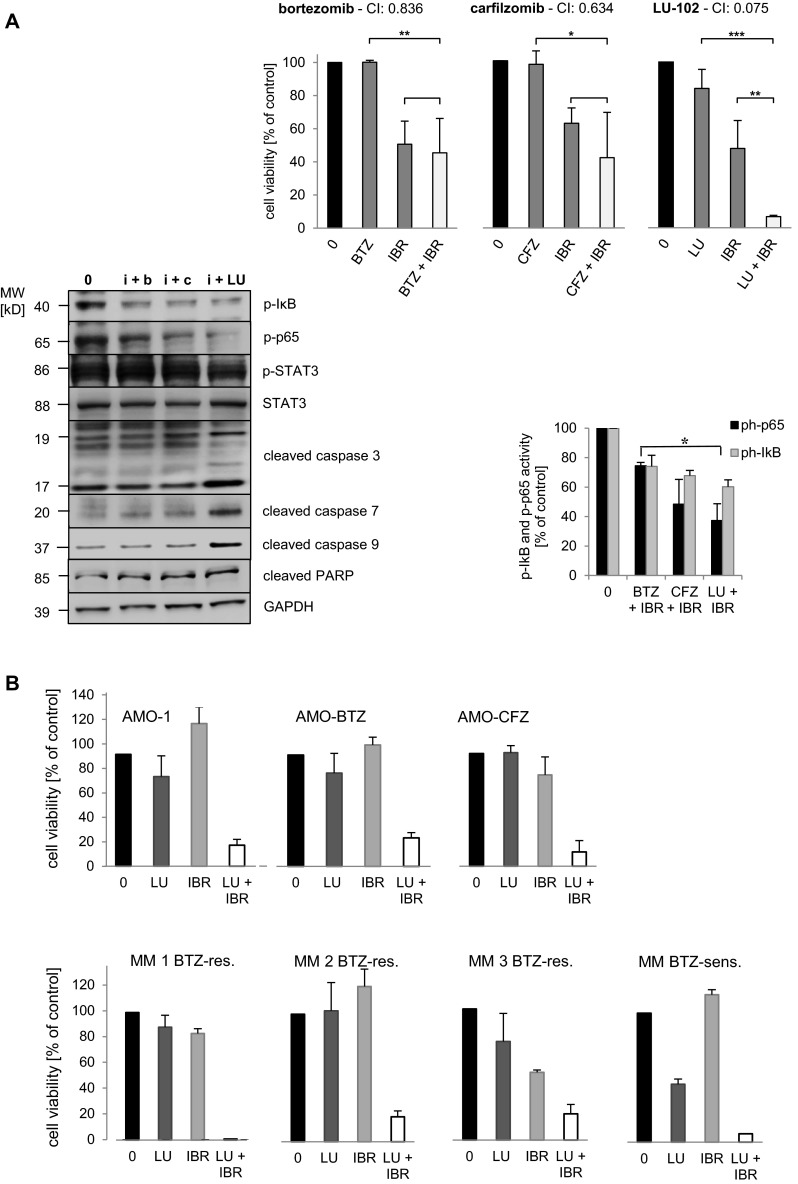
Cytotoxic effect of ibrutinib in combination with bortezomib, carfilzomib, and LU-102. **a** *Upper panel* INA-6 cells were incubated with bortezomib (BTZ) 3.3 nM, carfilzomib (CFZ) 3.3 nM, LU-102 (LU) 3.3 µM or ibrutinib (IBR) 10 µM or a combination of ibrutinib with one of the proteasome inhibitors, and cell viability was assessed. (*CI* combination index). Statistically significant differences were expressed as *p < 0.05, **p < 0.01, and ***p < 0.005. *Lower panel*, *left* After incubation of INA-6 cells with DMSO control (0), or a combination of ibrutinib (i) 10 µM and either bortezomib (b) 10 nM, carfilzomib (c) 10 nM or LU-102 (LU) 3.3 µM, Western blots against p-IκB, p-p65, p-STAT3, STAT3, cleaved caspases 3, 7, 9 and cleaved poly ADP ribose polymerase (PARP) proteins were performed and quantified as before. *Right* quantification of the Western blot signals retrieved for p-p65 and p-IκB. *Bar graphs* represent amounts of the detected p-p65 and p-IkB species under the different treatments, relative to baseline values with DMSO treatment. *Asterisk* indicates a statistically significant quantitative difference between BTZ- and LU-102-treated samples *p* < 0.05. **b** *Upper panel* AMO-1, AMO-BTZ, and AMO-CFZ cells were incubated with LU-102 and ibrutinib (AMO-1: LU-102 3.3 µM, ibrutinib 10 µM; AMO-BTZ: LU-102 10 µM and ibrutinib 10 µM; AMO-CFZ: LU-102 3.3 µM and ibrutinib 33.3 µM) or with the combination of both for 48 h and cell viability was assessed by MTT assay. *Lower panel* Primary MM cells from four myeloma patients with bortezomib-resistant (3 patients, BTZ-res.) or bortezomib-sensitive (1 patient, BTZ-sensitive) MM were exposed to bortezomib (BTZ) 10 nM or LU-102 (LU) 1 µM, ibrutinib (IBR) 10 µM, or the combination of the latter two for 48 h, and cell viability was assessed

We next analyzed the effects of ibrutinib in combination with the different proteasome inhibitors on cytotoxicity and activity of the NF-κB pathway in INA-6 MM cells. The combination of ibrutinib with either BTZ or CFZ at sub-cytotoxic concentrations did not significantly affect cell viability, compared with ibrutinib alone (Fig. 4a, upper panel), and resulted in borderline synergistic cytotoxic activity with combination indices of 0.6 and 0.8, respectively. By contrast, the combination of ibrutinib with sub-effective LU-102 resulted in a highly synergistic cytotoxic effect on INA-6 cells with a combination index of 0.075. While myeloma cell viability after treatment with ibrutinib in combination with BTZ or CFZ was 46 and 42 %, respectively, and thus in the same order of magnitude as after ibrutinib monotherapy, it was reduced to 7 % by combination treatment with LU-102. Consistent with this, as well as with the data described above, the combination of ibrutinib with LU-102 showed superior suppression of p-IκB, p-p65, and p-STAT3 protein expression, as well as induction of cleaved caspase 3, 7 and 9 and cleaved PARP, compared with ibrutinib in combination with either bortezomib or carfilzomib (Fig. 4a, lower panel). These findings demonstrate that that LU-102 is a superior combination partner for ibrutinib, compared with CFZ or BTZ, to induce cytotoxicity in MM cells.

### Ibrutinib in combination with LU-102 shows synergistic cytotoxicity against proteasome inhibitor-resistant MM cells

We next explored to what extent the combination of LU-102 and ibrutinib may likewise be cytotoxic against MM cells resistant against BTZ or CFZ. To serve as a model for proteasome inhibitor resistance, AMO-1 MM cell lines were adapted to grow in the presence of the two approved proteasome inhibitors as published before for bortezomib, yielding AMO-1 cell populations with differential sensitivity for BTZ or CFZ (AMO-1, AMO-BTZ, AMO-CFZ, supplemental material). Interestingly, CFZ-adapted cells (AMO-CFZ) were still sensitive to BTZ at high concentrations, while BTZ-adapted cells (AMO-BTZ) lacked CFZ sensitivity up to 100 nM, suggesting different mechanisms of proteasome inhibitor resistance between these two cell populations or the two drugs. When these cell populations were exposed to ibrutinib or LU-102 at sub-effective concentrations or to the combination of both drugs, respectively, we observed strong synergistic cytotoxic activity of this combination against the AMO-1 wild-type cells as well as the CFZ- or BTZ-adapted populations (Fig. 4b, upper panel and supplemental material). To further support that MM cells that are resistant to the approved proteasome inhibitors are sensitive to the combination of LU-102 and ibrutinib, we obtained primary MM cells from three patients with clinically bortezomib-resistant MM and exposed them to bortezomib 10 nM or LU-102 1 μM, ibrutinib 10 μM, or the combination of the latter two in vitro (Fig. 4b, lower panel). Cell viability was largely unaffected by either of the drugs alone, while the combination between LU-102 and IBR again yielded a highly synergistic cytotoxic effect between both compounds (CI 0.015).

### BTZ does not induce consistent, synergistic cytotoxicity with ibrutinib in MM and MCL cell lines, in contrast to CFZ and LU-102

**Fig. 5:**
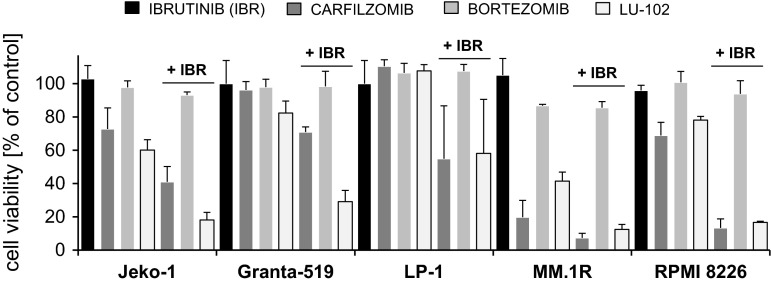
Cytotoxic effect of ibrutinib in combination with proteasome inhibitors. MCL cell lines (Jeko-1 and Granta-519) and MM cell lines (LP-1, MM.1R and RPMI 8226) were incubated with ibrutinib 10 µM (IBR), bortezomib 10 nM (BTZ), carfilzomib 10 nM (CFZ) and LU-102 3.3 µM (LU), or a combination of ibrutinib with one of the proteasome inhibitors for 48 h, and cell viability was assessed by MTT test. Results are expressed relative to cells treated with DMSO (0)

We finally challenged a panel of MM cell lines (LP-1, MM.1R, RPMI 8226) and MCL cell lines (Granta-519 and Jeko-1), which differed in their degree of BTK expression and ibrutinib sensitivity, with sub-effective concentrations of BTZ, CFZ, or LU-102, in the presence or absence of ibrutinib (Fig. 5), and calculated the respective combination indices for synergy (CI) after assessment of cytotoxicity by MTT assay. We observed that, in particular, BTZ was a poor combination partner with ibrutinib that did even result in an antagonistic CI of >1 in 4/5 of the cell lines tested (Table 1, median CI 4.9, range 0.3–30.3). By contrast, synergistic cytotoxicity with ibrutinib was seen in 5/5 cell lines with CFZ (median CI 0.12, range 0.03–0.22) and in 5/5 cell lines with LU-102 as combination partner (median 0.02, range 0.001–0.14). Our results therefore demonstrate that BTZ is a relatively poor combination partner to increase the cytotoxic effect of ibrutinib against MM or MCL cells, while CFZ, and in particular LU-102, show consistently strong synergistic cytotoxicity with ibrutinib. LU-102 appears to have an even stronger synergistic potential in combination with ibrutinib than CFZ, based on the CI values calculated.

**Table 1:** Combination Index (CI) for cytotoxic activity, MTT test

	BTK protein	IBR + BTZ	IBR + CFZ	IBR + LU-102
Granta-519 (MCL)	++	5.109	0.099	0.036
Jeko-1 (MCL)	–	4.994	0.144	0.015
LP-1 (MM)	++	30.333	0.031	0.001
MM.1R (MM)	+	4.319	0.220	0.145
RPMI 8226 (MM)	–	0.300	0.032	0.023
INA-6 (MM)	+++	0.83	0.63	0.075
Median		4.9	0.12	0.02

Combination indices (CI) for synergistic cytotoxic activity in vitro (Fig. 5) between ibrutinib (IBR) in combination with either bortezomib (BTZ), carfilzomib (CFZ), or LU-102 were calculated from MTT tests as described in materials and methods using the MCL and MM cell lines indicated. A CI < 0.8 indicates synergism, a CI > 1 antagonism. The different degrees of expression of BTK protein are indicated for the respective cell lines, as assessed by Western blot (– for no detectable BTK protein, +, ++, +++ for little, moderate and strong BTK protein expression, respectively)

**Fig. 6:**
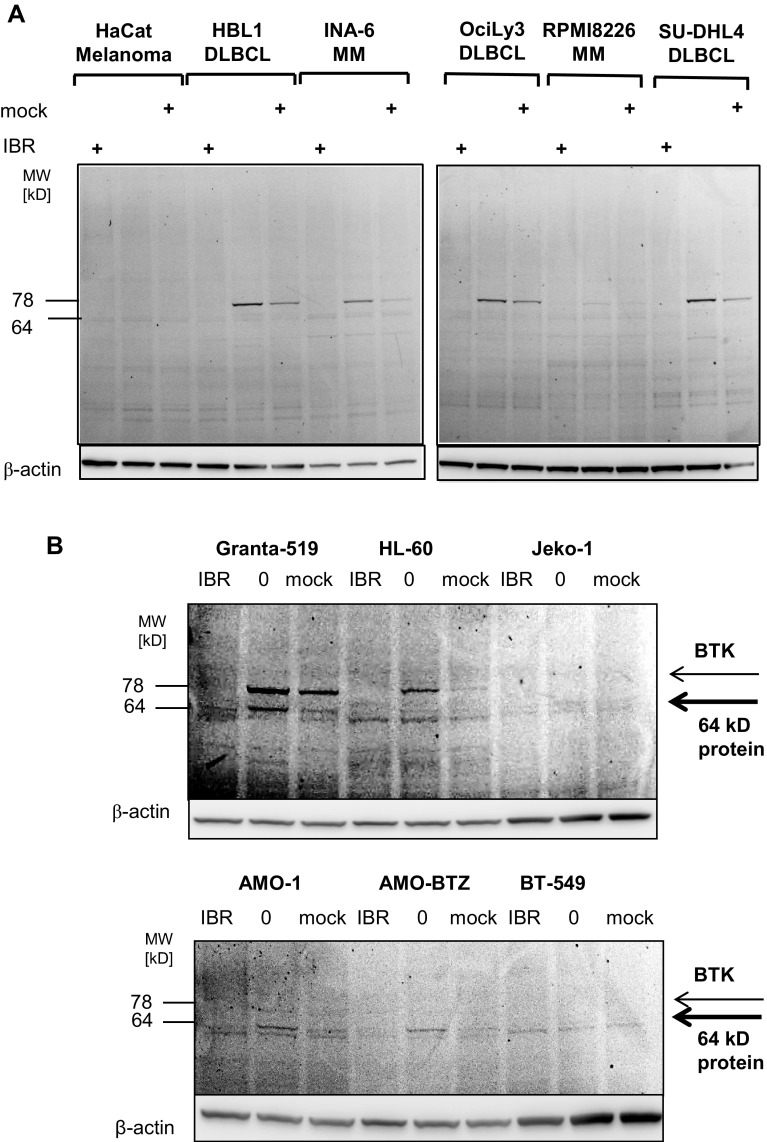
Visualization of ibrutinib-targeted proteins using a fluorescent synthetic ibrutinib-analog as activity-based covalent problem. **a** DLBCL type cell lines (HBL1, OciLy3, SU-DHL4), MM (INA-6, RPMI 8226) and the HaCat melanoma cell line were incubated with either DMSO, ibrutinib (IBR), or a mock version of ibrutinib-ABP lacking the active warhead (mock, 1 µM each), before cells were exposed to activity-based covalent affinity labeling (1 μM) by incubation with ibrutinib-ABP. After cell lysis, cellular protein was dissolved by SDS-PAGE and fluorescent signals visualized using a fluorescence reader. Western blotting for β-actin served as loading control from the same samples. **b** BTK-expressing (Granta-519, HL-60) cell lines and cell lines without BTK expression (Jeko-1, AMO-1, AMO-BTZ, as well as the breast cancer cell line BT-549) were incubated with DMSO (0), ibrutinib (IBR), or a mock treated as before, and ibrutinib-reactive protein species were visualized as before. Western blotting for β-actin served as loading control

### Visualization of a non-BTK off-target protein of ibrutinib

Interestingly, the synergistic cytotoxic activity between ibrutinib and LU-102 was observed not only in MM/MCL cells lines with high BTK expression, but to almost the same extent also in cells with low/absent detectable BTK protein expression. This suggested that ibrutinib may have additional, as yet unknown cellular targets.

To identify putative additional cellular targets for ibrutinib, we synthesized a cell-permeable derivative of ibrutinib with an incorporated fluorescent label that can be used to tag and visualize ibrutinib-reactive proteins in intact cells after cell lysis and SDS-PAGE due to the covalent target binding of the active compound [27]. Intact cells were incubated with this activity-based ibrutinib probe (ibrutinib-ABP) and subjected to subsequent cell lysis, SDS-PAGE, and fluorescence in-gel detection (Fig. 6). To verify the ibrutinib-sensitive nature of binding of the probe and the specificity of the labeled polypeptides, control cells were pre-incubated either with ibrutinib or with an inactive version of the probe that lacked the reactive group for covalent target binding (mock). Active BTK was identified as prominently labeled protein species in BTK-positive cells, based on its expected MW of 78 kD, and the nature of labeling that could be blocked by preincubation with ibrutinib but could only be partly competed by the non-covalent mock competitor of the affinity label. Also as expected, and consistent with our results from Western blots, INA-6 cells showed a robust BTK signal, while this signal was considerably lower in RPMI 8226, AMO-1, AMO-BTZ, and Jeko-1 cells, as well as in non-B-cell-type samples. Treatment of INA-6 cells with 1 mM ibrutinib entirely abolished the BTK activity signal in the absence of significant cytotoxicity (see Fig. 1b, indicating that BTK-expressing MM cells may not undergo significant cytotoxicity upon BTK inhibition alone.

We observed a second protein species of lower labeling intensity at 64 KD (upper band of the doublet polypeptides at 61 and 64 kD), which was decorated by the probe in specific way, comparable to BTK labeling, and whose labeling was antagonized by ibrutinib or, to a lesser extent, the non-covalent label with selectivity similar to that of BTK. This protein was detectable with low, but reproducible signal intensity in all B-cell lines used, irrespective of their BTK expression, and in particular in AMO-1 and AMO-BTZ cells. This labeled 64-kD polypeptide was absent from BT-549 breast cancer cells. We hypothesize that this protein is likely an as yet unidentified target kinase of ibrutinib distinct from BTK that may be the relevant for the effects of ibrutinib on BTK-negative MM and MCL cells alone or in combination with proteasome inhibition.

## Discussion

Our data demonstrate that LU-102 has superior synergistic cytotoxic activity with ibrutinib on MM and MCL cells, compared to BTZ, most likely due to its synergistic inhibition of p-IκB and the canonical NF-κB pathway, and that the combination of ibrutinib and LU-102 can overcome BTZ and CFZ resistance in vitro. We furthermore provide a first example that PI that target different proteasome active subunits can differentially affect cancer cell signaling or the activity of targeted cancer therapies: β2-selective proteasome inhibition did not result in gross quantitative inhibition of proteolytic degradation, as seen by the absence of accumulating polyubiquitinated protein after LU-102 treatment, but reduced p-IκB levels and was more effective in mediating cytotoxicity in combination with ibrutinib, compared with bortezomib that is exclusively inhibiting β5/β1 activity (Fig. 3). This has implications for the development of future proteasome-inhibiting therapies and drugs, where to date little attention is being paid to the different patterns of inhibition of the total of six active subunits of the constitutive and immuno-proteasome.

The potential use of proteasome inhibition in combination with the BTK inhibitor ibrutinib (IBR) in MM has already entered clinical trials (ClinicalTrials.gov, NCT01962792), although we currently lack understanding of the molecular interactions between both targeted pathways. Moreover, proteasome inhibitors with different active groups and molecular targets continue to be developed, while we have little rationale that guides our choice of proteasome inhibitor selected for such a strategy. Based on published information, we predicted that bortezomib may partially counteract the molecular mechanism of action of ibrutinib in MM cells, because it induces NF-κB activation in MM cells [18], while ibrutinib acts, at least partially, through NF-κB inhibition in MM [26].

The molecular basis for the different effects of bortezomib and carfilzomib, on the one hand, and LU-102 on the other hand, on the p-IκB/IκB balance remains to be explained. Consistent with our data from INA-6 cells, Hideshima demonstrated in RPMI 8226 myeloma cells, that not only bortezomib, but also the β5-directed, reversible peptide aldehyde proteasome inhibitor MG-132 and the irreversible pan-proteasome inhibitor lactacystin resulted in accumulation of p-IκB and decreased IκB levels which are predicted to counteract the molecular activity of ibrutinib. Thus, neither the nature of the chemical warhead of the proteasome inhibitor (peptide aldehyde, epoxyketone, β-lactone, and petide boronate), nor its reversibility of target binding seems to be of importance for these differential effects. In contrast to the proteasome inhibitors mentioned above, LU-102 does not inhibit or co-inhibit the rate-limiting β5 subunit of the proteasome, but provides β2-selective proteasome inhibition. This β2-selective proteasome inhibition results in a less effective quantitative inhibition of proteasomal proteolysis, as demonstrated by the absence of accumulated polyubiquitinated protein in our results. Because p-IκB is a known proteasome substrate that undergoes proteasomal degradation, we speculate that the increase in p-IκB after carfilzomib/bortezomib treatment may be attributed to the quantitative inhibition of protein destruction by these β5-inhibiting agents, in contrast to LU-102.

Recent data show that β5-directed proteasome inhibition can result in IκBα degradation via the lysosomal pathway that is induced in an IκB-kinase-independent manner [28]. Our results are consistent with the model that p-IκB-independent lysosomal degradation of IκBα occurs in MM cells exposed to β5-targeted proteasome inhibitors like carfilzomib or bortezomib. By contrast, because the β2-directed inhibitor LU-102 does not quantitatively affect the global balance between protein biosynthesis and destruction, LU-102 may not activate the lysosomal system to bypass the obstructed proteasomal proteolysis. Indeed, it has been demonstrated that blocking autophagy prevents bortezomib-induced NF-κB activation by reducing IκBα degradation in lymphoma cells [29].

Ibrutinib can increase the cytotoxic effect of bortezomib on myeloma patient cells in vitro [30], consistent with our results. However, we lack information about the nature of this effect, whether it is synergistic or may even in part be antagonistic, i.e., less than additive. The latter could still result in an increased cytotoxicity, compared with one drug alone, but would argue against further clinical development of both drugs in combination. In our results, bortezomib was not synergistic with ibrutinib in myeloma or mantle cell lymphoma cell lines (CI median 4.9), while carfilzomib showed a consistent synergism (CI median 0.12), in agreement with recent data from combinatorial drug screening in mantle cell lymphoma [31]. The most robust synergistic cytotoxicity, however, was induced by LU-102 (CI median 0.028, range 0.001–0.14), consistent with our analysis of p-IκB/IκB signaling, in multiple cell lines of MM and MCL as well as in bortezomib-resistant primary MM cells. Interestingly, this synergistic cytotoxic activity can be observed not only in MM/MCL cells lines with high BTK expression, but to almost the same extent also in cells with low/absent detectable BTK protein expression. This suggested that ibrutinib may have additional, as yet unknown cellular targets that may be involved in mediating this effect.

At present, it is unclear how inhibition of the β2 proteasome activity fosters synergistic cytotoxicity with ibrutinib. In contrast to bortezomib, also carfilzomib has some β2-inhibiting activity, which may provide the mechanistic link for the higher degree of synergy of carfilzomib or LU-102 with ibrutinib, compared with bortezomib. The proteasome is involved in controlling the intracellular levels of not only structural, but also most regulatory cellular proteins involved in cell cycling, proliferation, and differentiation. The different substrate specificities of the β5 versus β2 are consistent with the hypothesis that β5 inhibition versus β2 inhibition may differentially affect the turnover of some key regulatory proteins. A key role of individual “unlocking” proteases that can control the proteolytic destruction of individual substrate proteins in a multiprotease proteolytic pathway has been demonstrated for lysosomal antigen processing [32]. The β2 proteasome subunit may likewise control the degradation of a regulatory key protein that is involved in mediating ibrutinib-induced cell death.

Overcoming proteasome inhibitor resistance is a major problem for MM therapy. Recent data from thorough analysis of in vitro models and patient material demonstrate that proteasome inhibitor resistance is most likely a selection process of myeloma cells under the selective pressure of proteasome inhibitors. During this process, myeloma cells with preplasmoblast-like features and low levels of XBP-1, the major regulator of the unfolded protein response that at the same time determines plasmoblast differentiation and proteasome inhibitor sensitivity, accumulate [13]. Combining proteasome inhibition with a targeted therapy directed against the entire B-cell lineage, including immature cells, such as BTK inhibition, is a rational strategy to simultaneously treat both, the proteasome inhibitor-sensitive mature myeloma cells and the proteasome inhibitor-resistant immature cell populations. We here investigate this concept in an in vitro model that reflects these fundamental principles: The bortezomib-resistant and carfilzomib-resistant AMO-1 cells (AMO-BTZ, AMO-CFZ) used here have been generated under the selective pressure of the proteasome-inhibiting drugs and show significantly decreased XBP-1 levels, compared with the AMO-1 parental line, but at the same time lack mutations in the active sites of the proteasome ([10] and M. Kraus, unpublished results), so that they in this respect closely mimic the situation encountered in vivo. Compared to their parental cells, AMO-BTZ in particular expresses increased activity of the β2 proteasome subunit that is not targeted by bortezomib, a feature that might also contribute to BTZ resistance [10]. Our results demonstrate that combination treatment with the β2 inhibitor LU-102 and ibrutinib overcomes the features of proteasome inhibitor resistance and has significant synergistic cytotoxic activity against proteasome inhibitor-adapted myeloma cells, comparable to the proteasome inhibitor-sensitive parental cell line. Importantly, this feature was similarly observed when primary multiple myeloma cells from a patient with bortezomib-refractory disease were challenged with LU-102 in combination with ibrutinib, while both agents alone had no cytotoxic effect. It has been previously shown that the co-administration of ibrutinib with bortezomib increased cytotoxicity in bortezomib-resistant MCL and DLBCL cells; however, the NF-kB inducing effet of bortezomib has only been observed in MM and may be linked to the highly developed protein biosynthesis machinery of MM cells [18]. Murray et al. [33] very recently demonstrated that BTK activity was enhanced in bortezomib-resistant MM, and that co-treatment of MM cells with ibrutinib or a p65-targeted lentiviral construct and bortezomib can partly restore bortezomib sensitivity, highlighting the crucial role of NF-kB activity in bortezomib-resistant MM. Our results support this concept and furthermore show that ibrutinib in combination with the β2-selective PI LU-102 results in superior inhibition of NF-kB activation and significantly improved synergistic cytotoxicity, compared with bortezomib. The combination of ibrutinib and β2-selective proteasome inhibition may therefore represent a rational strategy to overcome proteasome inhibitor resistance of MM. At the same time, our results suggest therapeutic potential also for proteasome inhibitors that do not target the rate-limiting β5 proteasome subunit.

Although our data strongly support synergistic cytotoxicity between LU-102 and ibrutinib on myeloma cells, involving at least partly the NFkB pathway, BTK is likely not the only relevant target of ibrutinib in this setting. In our results, as well as other studies investigating the cytotoxic activity of ibrutinib on myeloma cells in vitro [26, 30], only very high concentrations of ibrutinib (10 μM) triggered a direct cytotoxic effect on myeloma cells, and BTK-negative cells were sensitive for synergistic cytotoxicity with ibrutinib and LU-102, suggesting alternative targets that are likewise inhibited by ibrutinib in myeloma cells. Indeed, ibrutinib (formerly PCI-32765) is known to target >20 different kinases at IC50 values below 1 μM [34]. Importantly, the cytotoxic activity of ibrutinib in myeloma has meanwhile been confirmed in a clinical phase II trial, but also clinically the ibrutinib dose required for maximum clinical mono-activity against myeloma was twice the standard dose used in CLL (840 mg daily, Vij R, et al. ASH 2014. Abstract 31), also suggesting alternative targets with slightly lower affinity involved in ibrutinib activity in myeloma. Using a fluorescence-labeled derivative of ibrutinib, we here demonstrate that indeed ibrutinib a non-cytotoxic concentration of 1 μM not only eliminates BTK activity in MM cells, but specifically targets also an unidentified 64-kD polypeptide that is present also in bortezomib-resistant myeloma cells that lacked detectable BTK expression but were sensitive to the combination ibrutinib + LU-102. We are currently on the way to design a biotin derivative of this ibrutinib probe to be able to isolate and identify the 64-kDa protein species and to prove its functional importance. At current stage, our data suggest that ibrutinib has additional, non-BTK targets in myeloma cells, which may be of functional relevance for the mechanism of action of ibrutinib against myeloma.
